# Clinical utility of overviews on adverse events of pharmacological interventions

**DOI:** 10.1186/s13643-023-02289-z

**Published:** 2023-07-31

**Authors:** Thilo Sachse, Salmaan Kanji, Pierre Thabet, Sven Schmiedl, Petra Thürmann, Fadi Guirguis, Shellyza Sajwani, Marie-France Gauthier, Carole Lunny, Tim Mathes, Dawid Pieper

**Affiliations:** 1 Faculty of Health, School of Medicine, Institute for Research in Operative Medicine, Witten/Herdecke University, 51109 Cologne, Germany; 2 The Ottawa Hospital and Ottawa Hospital Research Institute, Ottawa, Ontario Canada; 3 Hôpital Montfort and University of Ottawa, Ottawa, Ontario Canada; 4 Philipp Klee-Institute of Clinical Pharmacology, Helios University Hospital Wuppertal, Witten/Herdecke University, Chair of Clinical Pharmacology, Wuppertal, Germany; 5 The Ottawa Hospital, Ottawa, Ontario Canada; 6 Knowledge Translation Program, Unity Health Toronto and the Cochrane Hypertension Group, St. Michael’s Hospital, University of British Columbia, Vancouver, Canada; 7 Institute for Medical Statistics, University Medical Center Göttingen, Göttingen, Germany; 8 Faculty of Health Sciences Brandenburg, Brandenburg Medical School (Theodor Fontane), Institute for Health Services and Health System Research, Rüdersdorf, Germany; 9 Center for Health Services Research, Brandenburg Medical School (Theodor Fontane), Rüdersdorf, Germany

**Keywords:** Overviews, Meta-reviews, Adverse events, Clinical utility, Pharmacology, Evidence synthesis

## Abstract

**Background:**

Overviews (i.e., systematic reviews of systematic reviews, meta-reviews, umbrella reviews) are a relatively new type of evidence synthesis. Among others, one reason to conduct an overview is to investigate adverse events (AEs) associated with a healthcare intervention. Overviews aim to provide easily accessible information for healthcare decision-makers including clinicians. We aimed to evaluate the clinical utility of overviews investigating AEs.

**Methods:**

We used a sample of 27 overviews exclusively investigating drug-related adverse events published until 2021 identified in a prior project. We defined clinical utility as the extent to which overviews are perceived to be useful in clinical practice. Each included overview was assigned to one of seven pharmacological experts with expertise on the topic of the overview. The clinical utility and value of these overviews were determined using a self-developed assessment tool. This included four open-ended questions and a ranking of three clinical utility statements completed by clinicians. We calculated frequencies for the ranked clinical utility statements and coded the answers to the open-ended questions using an inductive approach.

**Results:**

The overall agreement with the provided statements was high. According to the assessments, 67% of the included overviews generated new knowledge. In 93% of the assessments, the overviews were found to add value to the existing literature. The overviews were rated as more useful than the individual included systematic reviews (SRs) in 85% of the assessments. The answers to the open-ended questions revealed two key aspects of clinical utility in the included overviews. Firstly, it was considered useful that they provide a summary of available evidence (e.g., along with additional assessments, or across different populations, or in different settings that have not been evaluated together in the included SRs). Secondly, it was found useful if overviews conducted a new meta-analysis to answer specific research questions that had not been answered previously.

**Conclusions:**

Overviews on drug-related AEs are considered valuable for clinical practice by clinicians. They can make available evidence on AEs more accessible and provide a comprehensive view of available evidence. As the role of overviews evolves, investigations such as this can identify areas of value.

**Supplementary Information:**

The online version contains supplementary material available at 10.1186/s13643-023-02289-z.

## Background

A rather new form of evidence synthesis used to summarize harms of interventions are overviews of reviews. While systematic reviews (SRs) try to collect, appraise, and synthesize evidence across multiple primary studies, overviews do the same across multiple SRs. They systematically identify, collect, and analyze data from SRs on the efficacy, effectiveness, or harms of healthcare interventions to provide easily accessible information for decision-makers [1]. The *Cochrane Handbook for Systematic Reviews of Interventions* defines different purposes for overviews [1], one of them being to “examine evidence about adverse effects of an intervention from two or more systematic reviews of use of an intervention for one or more conditions or populations,” which may “help identify and characterize the occurrence of rare events.” While overviews are sometimes also referred to as “umbrella reviews” or “(systematic) reviews of (systematic) reviews,” hereafter, we will use the term “overviews.”

The assessment of harms of pharmacological interventions is known to be challenging [2], particularly with respect to rare adverse events [3]. Different methodological approaches are used in research on harms, from spontaneous reporting systems to epidemiological study types [2]. These are known to have several drawbacks when it comes to comprehensively investigating harms of an intervention [2, 4]. Spontaneous reporting systems are used after intervention market approval and have important limitations such as underreporting and incapability to determine event rates [2]. Randomized controlled trials (RCTs) are typically designed to evaluate beneficial effects of drugs. The sample size of RCTs is usually too small to detect and quantify differences in particular in serious adverse outcomes because these are often rare or occur at a lower rate than efficacy outcomes [2]. SRs aim to evaluate the efficacy, effectiveness, and/or safety of healthcare interventions. Investigators have found that methodological steps recommended for investigating harms in SRs were often ignored, and that evidence on harms from SRs was unreliable [4, 5]. For example, SRs on harms of gabapentin reported differing results and conclusions despite similar identified sources of evidence [5]. These dissimilarities were attributed to the different selections of harm outcomes and different analytic approaches [4, 5]. Overviews investigating harms might help to address some of the aforementioned challenges.

Nomenclature for describing harms varies in the healthcare literature [2, 6]. A general term to describe harm is adverse event (AE). An AE is defined by the World Health Organization (WHO) as “any untoward medical occurrence that may appear during treatment with a pharmaceutical product but which does not necessarily have a causal relationship with the treatment” [7]. For simplicity and because of the broad definition, we will henceforth use the term “AE”.

Cochrane acknowledges that clinicians and decision-makers need a “friendly front end” to summarize evidence [8]. Overviews could potentially serve this purpose. For example, overviews could present results from inconsistent SRs on harms of the same interventions together with possible explanations for the inconsistencies. Hereby, overviews can help resolve and understand differences in results from multiple SRs on the same topic. Furthermore, by including multiple SRs on the same intervention, each with different indications, overviews might provide a more comprehensive view of harms associated with the intervention, which cannot be done in SRs limited to specific indications [4]. To the best of our knowledge, the perspective of clinicians towards the utility of overviews as a source of clinical information has never been formally investigated.

In a previous study, we identified 27 overviews exclusively focusing on AEs of pharmacological interventions and investigated their methodological and reporting characteristics [9]. We found that the methodological approaches authors used varied widely, and available guidance for the conduct of overviews was not used by many overview authors.

In this present study, we focus on content-related aspects of these overviews on AEs and aim to assess their clinical utility. By clinical utility, we mean the degree to which the research is useful in clinical practice, as perceived by clinical pharmacists and physicians specialized in clinical pharmacology. This definition is intentionally vague as clinical utility should be defined by the reader/knowledge user and can be highly subjective. In this context, an overview may provide more clinical utility than the included SRs individually.

## Methods

This study is a sub-study of a larger project that was described in an a priori published protocol [10].

### Derivation of overview sample

For this study, we used a set of 27 overviews exclusively investigating AEs of pharmacological interventions, as collected in a previous study [9]. This sample was obtained from a systematic search of MEDLINE, Embase, Epistemonikos, and the Cochrane Database of Systematic Reviews (CDSR) from database inception to May 17, 2021. Titles and abstract were screened for eligibility in duplicate (E. D., T. S.). Potentially eligible full texts then were obtained and assessed for their eligibility by two reviewers independently (D. P., T. S.). In case of disagreement, a third reviewer with clinical expertise (P. Th., P. T., S. K., S. Sch.) was consulted, and disagreements were resolved through discussion. A detailed description of the search strategy and study selection can be found in the larger project [9].

### Extraction of relevant data

Relevant data (e.g., data on population, intervention, comparator, outcome (PICO) studied in the overview, number and type of included studies, the primary research question as stated in the overviews) was extracted by one reviewer (T. S.) from the included overviews, and a second reviewer (D. P., T. M.) verified the results. Consensus was sought in case of discrepancies. A full list of extracted data can be found in an additional file (see Additional file 1).

### Development of assessment tool

For the assessment of clinical utility, we developed a tool containing a summary page with the extracted data for each overview. In addition, we developed a worksheet with four open-ended guiding questions and three statements on clinical utility. The tool was designed in collaboration with reviewers with methodological expertise on overviews (D. P., C. L., T. S., S. K., P. Th.) and reviewers with pharmacological expertise (P. Th., P. T., S. K., S. Sch.). Prior to the actual assessment, four reviewers with pharmacological expertise (P. Th., P. T., S. K., S. Sch.) tested the tool on one of the included overviews and provided feedback. The overview [11] was chosen against the background that all pharmacological experts had sufficient expertise with the clinical topic. The piloting feedback was used to modify the tool. According to the test feedback, only minimal modification was required. The assessment tool can be found in an additional file (see Additional file 2). Using this format, we created a separate, customized file for the assessment of each included overview.

### Identifying clinical experts

Assessors were required to be clinical pharmacists or physicians specialized in clinical pharmacology with sufficient level of knowledge and clinical expertise related to the topics of the overviews to which they were assigned. We employed a convenience sampling approach for the recruitment of clinical experts to assess the clinical utility of included overviews. First, pharmacological experts who were already members of the project (S. K., P. T., P. Th., S. Sch.) were recruited to conduct the assessments of overviews with topics in their area of expertise. As the range of clinical topics within our set of overviews was broad, additional clinical experts (F. G., M. F. G., S. S.) were sought based on remaining overview topics from existing professional contacts. There was no financial compensation for participation.

### Assessment of clinical utility

For the assessment of clinical utility, the experts were provided with the full paper and an assessment file (which included extracted key PICO elements) for each of their assigned overviews. They were asked to provide comments on each guiding question and to rank their agreement with the provided statements as “strongly disagree,” “somewhat disagree,” “somewhat agree,” or “strongly agree” within the Microsoft Word file. For the comments on the open-ended questions, there were no specifications regarding the length of the comments. The purpose of the ranking of statements was to determine the clinical utility of each overview, while the open-ended questions aimed to determine how utility was achieved in the overviews. The assessments were performed from May 6, 2022, to June 24, 2022.

### Analysis

We calculated and report frequencies for the ranking of utility statements. We used Microsoft Excel to categorize the comments on the open-ended questions qualitatively. We used a multilevel inductive thematic analysis [12] to derive aspects of clinical utility in overviews from the comments. We did not apply any preconceived themes but developed the categories from the comments. First, one reviewer (T. S.) read the answers to the questions and derived main categories of aspects of clinical utility from the meaning of the answers (e.g., “The findings consolidate previous knowledge from other studies” was categorized as “Summary of SR results”). Second, the same reviewer reread the answers and derived subcategories, if possible (e.g., “each individual systematic review studied varying mutations, and it was useful to review an overview article that providing a holistic viewpoint” was categorized as “Summary of results that were not synthesized or presented in a single SR”). During the analysis, the existing categories were constantly reviewed and adjusted if necessary to ensure consistency. A second reviewer (D. P.) verified the results.

## Results

### General characteristics of included overviews

We included 27 overviews in this study [11, 13–38]. The included overviews focused on several clinical domains with oncology and psychiatry being the most frequent. The interventions examined in the overviews ranged from specific substances (e.g., Irinotecan [17]), to classes of drugs (e.g., proton-pump inhibitors [13]) to general types of pharmacological interventions (e.g., vaccines [28]). AE outcomes of interest ranged from specific prespecified outcomes (e.g., weight change [24]) to not being restricted at all [21]. Full details on the characteristics of the included overviews can be found in an additional file (see Additional file 3).

### Recruitment of clinical experts

In total, seven clinical experts were recruited to perform the assessments of clinical utility (P. Th., P. T., S. K., S. Sch., F. G., M. F. G., S. S.). Of these, five are clinical pharmacists (P. T., S. K., F. G., M. F. G., S. S.), and two are physicians specialized in clinical pharmacology (P. Th., S. Sch.). Details of the experts can be found in an additional file (see Additional file 4). Due to resource limitations, each overview was assessed by one reviewer.

### Utility assessment

The overall agreement with the provided statements was high (Table 1). For each overview, at least one of the statements was “somewhat agreed.” For 15/27 (56%), each statement was at least “somewhat agreed” [11, 17, 18, 22, 23, 25, 26, 28–32, 34, 36, 38], and in 7/27 (26%) assessments, each statement was “strongly agreed” [11, 18, 23, 25, 26, 29, 32]. The ranking of agreement for each overview can be found in an additional file (see Additional file 3).

**Table 1:** Agreement with assessment statements (*N* = 27)

**Statements**	**Frequency (%)**
**This overview generates new knowledge not previously known from existing systematic reviews**	
Strongly agree	9 (33)
Somewhat agree	9 (33)
Somewhat disagree	8 (30)
Strongly disagree	1 (4)
**This overview adds value to the existing literature on this topic**	
Strongly agree	10 (37)
Somewhat agree	15 (56)
Somewhat disagree	2 (7)
Strongly disagree	0 (0)
**This overview would be useful to clinicians when compared to the individual systematic reviews included in the overview**	
Strongly agree	7 (26)
Somewhat agree	16 (59)
Somewhat disagree	4 (15)
Strongly disagree	0 (0)

### Key aspects of clinical utility

Analysis of the answers to the open-ended questions revealed two key aspects of clinical utility of overviews on AEs, as well as several other aspects that were positively mentioned in the assessments but were not necessarily related to the overview methodology (Table 2). A detailed list of the answers and their categorization can be found in an additional file (see Additional file 5).

**Table 2:** Key aspects of clinical utility in overviews on adverse events emerging from the assessments

**Summary of SR results**
• Summary contrasting inconsistent SR results ▪ Example: “Most of the individual SRs and meta analyses offer important, even though sometimes contradictory, information. So, it is nice to have the most relevant outcomes and figures in an overview” (Uguz 2020)
• Summary demonstrating consistent SR results ▪ Example: “It provides a more extensive view of the published data and confirms that the data overall is consistent” (Thulliez 2018)
• Summary of results that were not synthesized or presented in a single SR ▪ Example: “There is value to this overview given that it consolidates knowledge on adverse effects of bisphosphonate from 8 systematic reviews that each looked at only one adverse effect. This overview provides a comprehensive understanding of adverse events of bisphosphonates and not just the risk of one adverse event as done with prior systematic reviews” (Lu 2019)
• Summary of SR results with additional assessments (e.g., methodological quality assessments, assessments of the certainty of the evidence) ▪ Example: “There is new knowledge in this overview. This publication offers a summarized classification of evidence from 113 systematic reviews (SRs). This classification is based on terminology by the International Association for Research on Cancer, such as “sufficient systematic review evidence” of a specific hazard to human health. This classification was done after an appraisal of methodological quality of every systematic review based on the AMSTAR tool (A MeaSurement Tool to Assess systematic Reviews)” (Van Leeuwen 2020)
**New meta-analysis**
• New meta-analysis across conditions not analyzed together in included SRs ▪ Example: “The authors are interested in the prevalence of gastrointestinal and behavioral adverse events associated with steroid use in children with respiratory disease. The authors claim that AEs are poorly described in the 7 included SRs. In fact, they felt that they were so poorly described that they extracted the outcomes from primary studies and did their own meta-analysis. The output from this meta-analysis would not be found in any of the included SRs. The other source of added value here is that the overview authors’ research question was broad with respect to the diseases that children have that might require steroid therapy. There seems to be no SR asking this same question so the authors of this overview included SRs on a variety of respiratory disease for which steroid therapy was assessed. While this approach would definitely introduce unnecessary heterogeneity for efficacy outcomes, it would be reasonable to assume that AEs to steroids are independent of the disease allowing for the pooling of steroid trials of different respiratory diseases (i.e., asthma, pneumonia, croup, bronchiolitis)” (Fernandes 2014)
• New meta-analysis for subgroups or specific research questions not analyzed in included SRs ▪ Example: “New analyses have been conducted focusing on pediatric studies included as part of formerly published SRs (without age restrictions). Furthermore, additional pediatric studies were identified by updated searches” (Cates 2012)
• New meta-analysis increasing confidence in the results by including a higher number of patients than the included SRs ▪ Example: “Increasing evidence due to a higher number of patients used for calculating risk estimates” (Els 2017)
**Other aspects (that are not necessarily related to the overview methodology)**
• Highlighting gaps or limitations in current evidence
• Providing information on definitions of AEs, dose, duration, areas for improvement in future research
• Interesting or important results (e.g., differences in the timing of AEs in different populations)
• Structured presentation of results
• Useful approach (e.g., formulation of research question, choice of AE outcomes of interest, included primary study type, providing expert opinion, stating most rigorous included SR, estimation of number of needed participants to detect true difference in events rates, providing new guidance)

Abbreviations: *SR* systematic review; *AE* adverse event; *AMSTAR* A MeaSurement Tool to Assess systematic Reviews

Firstly, the overviews were found to be an easily accessible summary or consolidation of existing SR results. This aspect was mentioned in 19 of the assessments (19/27, 70%). Beyond the simple summary of existing data, this aspect was considered useful as it allowed inconsistent SR results to be contrasted or consistent SR results to be highlighted. Furthermore, it was mentioned as useful that overviews could summarize outcome data from multiple SRs that were not presented or synthesized in a single SR before, resulting in a comprehensive view of available evidence. AE rates attributed to a drug (or drug class) can be compared across different populations or treatment indications allowing for consolidation and comparison that may not be appropriate for a SR that focuses on efficacy as a primary objective. One feature that was particularly highlighted was the ability to present results from multiple SRs together with additional assessments that were performed by the overview authors (e.g., assessments of the methodological quality of included SRs, assessments of the certainty of the evidence). Through these additional qualitative assessments, overviews on AEs were found to generate new knowledge and to increase confidence in the results.

Secondly, assessors found utility when overviews reanalyzed data by conducting a new meta-analysis. This was mentioned in all assessments of overviews that performed such a reanalysis (5/5, 100%). The overviews were found to be able to analyze data across conditions that were not analyzed together before. Another purpose of new meta-analyses was to synthesize new data for specific subgroups or specific research questions. Furthermore, conducting a new meta-analysis was found to increase the confidence in the results as the number of patients synthesized in the analysis increased.

### Missing information in overviews on AEs

In addition, the assessments named aspects that were missing from the overviews, and that would have increased the clinical utility (Table 3). Moreover, other specific limitations of the included overviews were mentioned (these can be found in an additional file [see Additional file 5]). Missing details were mentioned in 18 assessments (18/27, 67%). The assessors criticized missing relevant information on the intervention (e.g., dosage, route of administration, duration of the treatment), on the population (e.g., age, pregnancy trimester), or on the clinical definitions of AEs. However, some assessments noted that it may not have been possible to obtain this information from the included SRs. Furthermore, in ten assessments (10/27, 37%), missing methodological steps were criticized (i.e., additional assessments (e.g., methodological quality assessments, assessments of the certainty of the evidence), new meta-analyses, or subgroup analyses).

**Table 3:** Missing aspects in the overviews that would increase clinical utility

**Missing further information**
• On the intervention (e.g., dosage, duration, co-medication, frequency) ▪ Example: “It would be useful to know more information about the specific dose of irinotecan used in these studies, to further examine the relationship between irinotecan dosing and UGT1A1 genotype” (Campbell 2016 Irinotecan) ▪ Example: “More importantly, not enough information is provided around duration of therapy or follow-up. Considering that many cancers take a long time to develop, knowing the duration of follow-up and SGLT2-I exposure is critical to understanding the relationship” (Pelletier 2020)
• On the population (e.g., age, pregnancy trimester, risk factors) ▪ Example: “As mentioned above, anticholinergic side effects are a major problem particularly for older patients. So, information about age would be helpful. Perhaps differences in the occurrence of these AEs between drugs are not drug-related but age-related (because of an older/younger population in the trials compared)” (Ozbilen 2009)
▪ On the outcome of interest (e.g., definitions of AEs, time of onset of AEs) ▪ Example: “Clinical definitions of neutropenia and grading of diarrhea would have increased clinical utility with regards to the extent of toxicity related to various mutation” (Campbell 2016 Fluoropyrimidine and Platinum-based chemotherapies)
• On study details (e.g., follow-up time) ▪ Example: “Duration of treatment, follow-up and time to onset of adverse events are not reported” (Pelletier 2021)
**Missing methodological steps**
• Additional assessments (e.g., methodological quality assessments, assessments of the certainty of the evidence) ▪ Example: “However, an assessment of overlap or discussion of the variability between SRs (discordance) would have been useful” (Abramowitz 2016)
• New meta-analysis ▪ Example: “Unfortunately, no statistical analysis was conducted (not even for ‘any infection’) due to the descriptive character of the overview” (Bonovas 2018)
• Subgroup analysis (e.g., for conditions, substances, dosage) ▪ Example: “It would be good if the rates of AE were further broken down by dose, specific steroid, route of administration, duration of use (they only included short term use defined as 2 weeks or less but many of these trials are single dose), and age of children (included neonates to 18 years of age)” (Fernandes 2014)

Abbreviations: *UGT1A1* uridine diphosphate glucuronosyltransferase-glucuronosyltransferase 1-1; *SGLT2-I* sodium-glucose transport protein 2 inhibitor; *AE* adverse event; *SR* systematic review

## Discussion

To our knowledge, this is the first study that systematically assesses clinical utility of overviews on AEs. We identified 27 overviews on AEs of pharmacological interventions. The assessments found that most overviews were considered clinically useful. We revealed several key aspects that were highlighted as useful if included and criticized if missing. We also identified information that was often missing from overviews on AEs but would increase clinical utility.

The assessments showed high levels of agreement with the statements we provided. In two out of three assessments, assessors agreed that the overviews generated new knowledge that was not previously known from the included SRs (67%). In the vast majority of assessments, assessors agreed that the overviews added value to the existing literature on the topic (93%), and that the overview would be more useful to clinicians than the included SRs (85%). This demonstrates that overviews investigating AEs can be useful on many levels in clinical practice and emphasizes their potential to provide clinicians and knowledge users with “friendly front-end” information, as proposed by Cochrane [8].

The only currently available guidance specifically related to overviews examining AEs is that from the *Cochrane Handbook of Systematic Reviews of Interventions* chapter on overviews [1], which states overviews can “examine evidence about adverse effects of an intervention from two or more systematic reviews of use of an intervention for one or more conditions or populations,” which may “help identify and characterize the occurrence of rare events.” There is no further guidance on how exactly this can be achieved in overviews investigating AEs.

Our assessments highlight two key aspects of clinical utility that can inform authors who wish to plan and conduct an overview on AEs: Firstly, the ability to summarize evidence from multiple SRs, and secondly, the possibility to conduct a new meta-analysis with existing data.

Overviews primarily summarizing evidence from the included SRs were found particularly useful if additional assessments (e.g., of methodological quality, primary study overlap, or of the certainty of the evidence) were conducted, and the results of these assessments were presented in the overviews. According to our previous investigation of the methodological approaches of the overviews included in this study, the majority of overviews conducted and presented the results of an assessment of methodological quality of included SRs (70%) [9]. Other assessments, such as the investigation of primary study overlap and an assessment of the certainty of evidence, which are both recommended in the Cochrane Handbook, were conducted much less frequently (22%, 33%) [9]. However, although the overviews were perceived as helpful, readers must be able to rely on these assessments. Therefore, they should be conducted appropriately to be reliable. Until there will be specific guidance on overviews of AE, overview authors should follow available guidance as outlined in the Cochrane Handbook [1]. Furthermore, summarizing evidence from the included SRs was found to be useful, because it provides the reader with aggregated information that could not have been presented in a single SR and otherwise would have to be extracted laboriously from individual SRs. Another ability of overviews on AEs that was found useful is to demonstrate consistent SR results or highlight and contrast inconsistent results, especially if the overview authors provided potential explanations for the different results within the discussion section.

In all assessments of overviews that have conducted a new meta-analysis, these new analyses were considered useful. This generally increases the number of participants and events, which increases the certainty of the results. In addition, the existing data can be used to answer specific research questions that have not been investigated so far (e.g., performing a meta-analysis of data on a specific subgroup). If authors planning an overview on AEs want to use existing data to answer a different question than the available SRs, they should consider extracting relevant data from SRs and analyzing it in a different way than the original analyses [1]. In this case, Cochrane recommends to reanalyze data that is reported within the included SRs using recommended standard meta-analytic methods (e.g., as described in the Cochrane Handbook [39]) [1]. Primary study data should only be obtained directly from the primary studies if not appropriately reported in the included SRs [1]. Therefore, before starting the actual overview, authors should consider how they will proceed if relevant data is missing from included SRs. Cochrane suggests noting the gap in coverage or extracting the relevant data directly from primary studies, enhancing comprehensiveness and rigor of overviews [1]. However, if the reporting of crucial data is poor in the SRs, the better option might be to conduct a new SR instead [1]. In addition, in the case of new primary studies, an update of previous SRs might be more appropriate. Another important issue to consider when conducting a new meta-analysis in overviews is potential bias. One important type of bias that is particularly relevant in studies on AEs is publication bias [40]. Our previous investigation of the methodology of overviews on AEs found that publication bias was discussed in only one of the 27 overviews. In general, handling publication bias in overviews is challenging as there are currently no established methods for this and authors have to rely on the information provided by the SRs. 

The assessors mentioned information missing from the overviews, such as details on the intervention or the studied population. However, it was noted that in many cases, it may not have been possible to obtain these data because they may not have been reported in the included SRs, which demonstrates the need for improved reporting in SRs on AEs [41–44]. In addition, many methodological shortcomings of RCTs (e.g., sample size too small, follow up too short) and SRs (e.g., overlooking guidance in the investigation of AEs) investigating and reporting AEs have been documented [2, 4, 5] and remain unchanged. To improve the investigation of AEs, changes in the conduct of RCTs and SRs have been proposed [4, 5, 45]. However, these issues cannot be resolved through overview methodology. Nevertheless, until improved methods for investigating AEs in primary studies and SRs are employed, overviews on AEs can be used to make existing evidence (albeit flawed) more apparent, accessible, or useful to knowledge users (e.g., clinicians, policymakers, or patients). Our assessment demonstrated that the overviews included in our study were found to be very informative and, in the majority, more useful than the individual SRs.

### Strengths and limitations

With this study, we provide a practical assessment of the utility of overviews on AEs. The assessment tool was developed in collaboration of experts in overview methodology with pharmacological experts. Through this and the assessment by pharmacological experts with clinical expertise, we provide the perspective of knowledge users on overviews, which has not been considered so far. The assessment tool uses a 4-point Likert scale. While this requires the assessors to choose between agreement and disagreement, it does not allow for neutral answers. Due to limited resources, there was only one assessor per overview, even though a duplicate assessment would have been preferable to reduce subjectivity. We did not record time taken to do an assessment. Furthermore, the assessment only allows conclusions to be drawn about whether the results of the overviews were considered useful. To ensure the reliability of the results of the overviews, their methodology and reporting should also be considered. We used a comprehensive search strategy to identify all eligible overviews, while we might still have failed to identify all. However, with a total of 27 overviews included in this study, and given that overview methodology can still be considered to be rather undeveloped, our findings need to be replicated in future. Study selection and data extraction were performed in duplicate.

## Conclusion

This study has shown that overviews on AEs are highly valuable for clinical practice. Overviews can make available evidence on AEs more accessible and provide a comprehensive view of available evidence. Additional assessments conducted by overview authors (e.g., of methodological quality, primary study overlap, certainty of the evidence) were found to increase the utility of overviews on AEs. Another purpose may be to generate new data on specific research questions that have not been explored in previous SRs. As the role of overviews evolves, investigations such as this can identify areas of value.

## Supplementary Information

**Additional file 1.** 

**Additional file 2.** 

**Additional file 3.** 

**Additional file 4.** 

**Additional file 5.**

## Data Availability

All data generated or analyzed during this study are included in this published article (and its supplementary information files).

## Abbreviations

AE: Adverse event
AMSTAR: A MeaSurement Tool to Assess systematic Reviews
CDSR: Cochrane Database of Systematic Reviews
PICO: Population, intervention, comparator, and outcome
RCT: Randomized controlled trial
SGLT2-I: Sodium-glucose transport protein 2 inhibitor
SR: Systematic review
UGT1A1: Uridine diphosphate glucuronosyltransferase-glucuronosyltransferase 1-1
WHO: World Health Organization
