# Supplementary material for: Clinical utility of overviews on adverse events of pharmacological interventions
Source: Syst Rev. 2023 Jul 31;12:131. doi: 10.1186/s13643-023-02289-z (PMC10388527; doi:10.1186/s13643-023-02289-z)
Supplement: Supplementary file 1 — Additional file 1. [file 13643_2023_2289_MOESM1_ESM.docx]

**Data extraction items**

- First author
- Year of publication
- Clinical domain
- Number of included systematic reviews
- Number of included primary studies
- Type of eligible primary studies
- Population
- Disease/condition
- Intervention: Group of drugs, substance(s)
- Comparator
- Adverse event outcome(s) of interest
- Primary research question (as stated in the last sentence or paragraph of the introduction- or background-section, plus information given elsewhere, if more meaningful)
