# Supplementary material for: Clinical utility of overviews on adverse events of pharmacological interventions
Source: Syst Rev. 2023 Jul 31;12:131. doi: 10.1186/s13643-023-02289-z (PMC10388527; doi:10.1186/s13643-023-02289-z)
Supplement: Supplementary file 2 — Additional file 2. [file 13643_2023_2289_MOESM2_ESM.docx]

**Assessment tool**

**Clinical utility of overviews (reviews of reviews) on adverse events of pharmacological interventions**

Overviews of reviews, sometimes called umbrella reviews or (systematic) reviews of (systematic) reviews, systematically identify, collect, and analyze data at the systematic review (SR)-level on the efficacy, effectiveness, and safety of healthcare interventions. They aim to provide easily accessible information for healthcare decision-makers. According to the Cochrane Handbook for Systematic Reviews of Interventions, one of the identified aims of overviews is to “*examine evidence about adverse effects of an intervention from two or more systematic reviews of use of an intervention for one or more conditions or populations*,” which may “*help identify and characterize the occurrence of rare events*.”

Since the methods used in overviews are evolving, there is not yet a consensus on what types of research questions involving adverse events are best answered using the overview methodology. In this study we aim to examine the clinical utility of published overviews on adverse events of pharmacological interventions. By clinical utility we mean the degree to which the research is useful in clinical practice. This definition is intentionally vague as clinical utility should be defined by the reader/knowledge user and can be highly subjective. In this context, an overview should provide more clinical utility than the included systematic reviews individually. In a previous study, we identified 27 overviews where the research question focuses on adverse events of pharmacological interventions. The interventions examined in the identified sample of overviews vary widely and therefore we are seeking the expert opinion of a variety of clinicians and other healthcare professionals.

We are inviting you as an expert in pharmacotherapy to help assess the clinical utility of these overviews. You have been specifically chosen for your clinical expertise and matched with an overview topic that is within your field. If you agree, we hope that your knowledge of the topic will allow you to comment on the clinical utility of the overview and how (if at all) it contributes to the existing knowledge on the topic. In addition to reading the overview, it may be beneficial for you (but not required) to scan the literature to get a sense of the state of the evidence at the time the overview was published.

We will provide the PDF of the article, a summary page with some basic data about the overviews, as well as the research question, and a worksheet to provide your assessment of clinical utility. There are no space or word limits. Please use as much space as you need to provide your assessment. The more information you provide about clinical context and why you made the assessment you did, the better we are able to understand your clinical perspective. After reviewing the worksheets, we may also reach out to you directly to discuss your opinions to ensure that we adequately represent your views.

We provide four guiding questions that can help you to judge the clinical utility of the overview:

- Did the overview generate new knowledge not previously established from existing systematic review data (i.e., does a well conducted overview provide additional value compared to the best systematic review included in the overview)?
- If there is new knowledge or additional value from the overview, how would you describe it? Perhaps data is consolidated across multiple systematic reviews of different populations providing a broader perspective. Perhaps data extracted from systematic reviews is analyzed in a way that has not been (or could not have been) done before in individual systematic reviews. Perhaps the overview addresses evidence from conflicting systematic reviews.
- As a knowledge user, would this overview be useful to clinicians, or would you have rather read the individual systematic reviews (or the best systematic review) included in the overview?
- Is there relevant information missing in the overview that would increase clinical utility (e.g., details on medications or dosage, definitions or diagnosis criteria of adverse events)?

For the assessment of the clinical utility of the included overviews, we ask you to provide open-ended answers to each of these questions. Afterwards we ask you to rank your agreement to three statements based on these questions as “strongly agree,” “somewhat agree,” “somewhat disagree,” or “strongly disagree.”

| **First author** |  |
| --- | --- |
| Year of publication |  |
| Clinical domain |  |
| No. of included SRs |  |
| No. of included primary studies |  |
| Type of eligible primary studies |  |
| Population, Disease/Condition |  |
| Intervention: Group of drugs, Substance(s) |  |
| Comparator |  |
| Adverse Event Outcome(s) of interest |  |
| Primary research question (as stated in the last sentence or paragraph of the introduction- or background-section, plus information given elsewhere, if more meaningful) |  |

**Clinical utility of overviews (reviews of reviews) on adverse events of pharmacological interventions
Summary page**

**Clinical utility of overviews (reviews of reviews) on adverse events of pharmacological interventions
Worksheet**

**Overview:**

1. **Did the overview generate new knowledge not previously known from existing systematic reviews (i.e., is there additional value compared to the best systematic review included in the overview)? Please explain.**
2. **If there is new knowledge, how would you describe the additional value from the overview? Please explain.**
3. **Would this overview be useful to clinicians or would you have rather read the individual systematic reviews (or the best systematic review) included in the overview? Please explain.**
4. **Is there relevant information missing in the Overview that would increase clinical utility (e.g., details on medications or dosage, definitions of adverse events)? Please explain.**
5. **Similar to the questions above, please rank your agreement with the following statements. Please mark your choices by changing the text to bold.**

| This overview generates new knowledge not previously known from existing systematic reviews. | Strongly Disagree | Somewhat Disagree | Somewhat Agree | Strongly Agree |
| --- | --- | --- | --- | --- |
| This overview adds value to the existing literature on this topic. | Strongly Disagree | Somewhat Disagree | Somewhat Agree | Strongly Agree |
| This overview would be useful to clinicians when compared to the individual systematic reviews included in the overview. | Strongly Disagree | Somewhat Disagree | Somewhat Agree | Strongly Agree |
