# Supplementary material for: Clinical utility of overviews on adverse events of pharmacological interventions
Source: Syst Rev. 2023 Jul 31;12:131. doi: 10.1186/s13643-023-02289-z (PMC10388527; doi:10.1186/s13643-023-02289-z)
Supplement: Supplementary file 3 — Additional file 3. [file 13643_2023_2289_MOESM3_ESM.docx]

**Characteristics of included overviews, rankings of agreement from assessments**

| **First author** | **Abramowitz [1]** |
| --- | --- |
| Year of publication | 2016 |
| Clinical domain | Gastroenterology |
| No. of included SRs | 26 |
| No. of included primary studies | Not reported |
| Type of eligible primary studies | Randomized controlled trials, observational studies, case series |
| Population, Disease/Condition | Adults with gastroesophageal reflux disease |
| Intervention: Group of drugs, Substance(s) | Proton-pump inhibitors |
| Comparator | Patients not receiving proton-pump inhibitor therapy |
| Adverse Event Outcome(s) of interest | Community-acquired pneumonia, enteric infections, bone fractures |
| Primary research question (as stated in the last sentence or paragraph of the introduction- or background-section, plus information given elsewhere, if more meaningful) | Introduction: “we performed an overview of systematic reviews and meta-analyses that address adverse events in PPI therapy, with the goal of providing clinicians with an unbiased, quantitative summary of adverse events to facilitate shared decisions when discussing PPI therapy with their patients.”  Methods: “The explicit research question—framed in a PICO format (ie, population, intervention, comparison, outcome)—was as follows: For patients with gastroesophageal reflux disease who received PPI therapy, what increase in adverse events— especially bone fractures, enteric infections, and communityacquired pneumonia (CAP)—was observed when compared with patients who did not receive PPI therapy? Our target population was adults, so studies limited to children were excluded.” |

| This overview generates new knowledge not previously known from existing systematic reviews. | Strongly Disagree | **Somewhat Disagree** | Somewhat Agree | Strongly Agree |
| --- | --- | --- | --- | --- |
| This overview adds value to the existing literature on this topic. | Strongly Disagree | **Somewhat Disagree** | Somewhat Agree | Strongly Agree |
| This overview would be useful to clinicians when compared to the individual systematic reviews included in the overview. | Strongly Disagree | Somewhat Disagree | **Somewhat Agree** | Strongly Agree |

| **First author** | **Bonovas [2]** |
| --- | --- |
| Year of publication | 2018 |
| Clinical domain | Gastroenterology |
| No. of included SRs | 10 |
| No. of included primary studies | Not reported |
| Type of eligible primary studies | Randomized controlled trials |
| Population, Disease/Condition | Patients with ulcerative colitis |
| Intervention: Group of drugs, Substance(s) | Biological agents: Adalimumab, Golimumab, Infliximab, Vedolizumab |
| Comparator | Not specified |
| Adverse Event Outcome(s) of interest | Infection (any, serious, opportunistic), tuberculosis, malignancies |
| Primary research question (as stated in the last sentence or paragraph of the introduction- or background-section, plus information given elsewhere, if more meaningful) | Introduction: “Given that meta-analysis of randomized controlled trials (RCTs) ranks high in the proposed hierarchy of evidence, an umbrella review of meta-analyses of RCTs on the safety of biologics in UC would be useful.”  Abstract: “the aim of this umbrella review was to effectively summarize the accumulated evidence from randomized controlled trials (RCTs) on the safety of biological therapies for UC into one accessible and usable document.” |

| This overview generates new knowledge not previously known from existing systematic reviews. | Strongly Disagree | **Somewhat Disagree** | Somewhat Agree | Strongly Agree |
| --- | --- | --- | --- | --- |
| This overview adds value to the existing literature on this topic. | Strongly Disagree | Somewhat Disagree | **Somewhat Agree** | Strongly Agree |
| This overview would be useful to clinicians when compared to the individual systematic reviews included in the overview. | Strongly Disagree | Somewhat Disagree | **Somewhat Agree** | Strongly Agree |

| **First author** | **Campbell [3]** |
| --- | --- |
| Year of publication | 2016 |
| Clinical domain | Oncology |
| No. of included SRs | 6 |
| No. of included primary studies | Not reported |
| Type of eligible primary studies | Not specified |
| Population, Disease/Condition | Patients with cancer |
| Intervention: Group of drugs, Substance(s) | Cytostatic agents: Fluoropyrimidine and Platinum-based chemotherapies  Exposure of interest: “any form of germline polymorphism (e.g., SNPs, short tandem repeats and deletions), whether detected by candidate gene analysis or genome-wide association study“ |
| Comparator | Not specified |
| Adverse Event Outcome(s) of interest | Any Fluoropyrimidine- or platinum-induced toxicities, adverse events or side effects |
| Primary research question (as stated in the last sentence or paragraph of the introduction- or background-section, plus information given elsewhere, if more meaningful) | Introduction: “in this report an ‘umbrella systematic review’; a systematic review of systematic reviews has been undertaken in order to identify and synthesize the findings of all systematic reviews that investigate the pharmacogenetics of FU or platinum-induced toxicity. The aim was to identify all genes, polymorphisms, populations and contexts where biomarkers for FU- or platinuminduced toxicity have been validated, and are potentially ready for clinical application as risk factors to add information to personalized medicine knowledge base.” |

| This overview generates new knowledge not previously known from existing systematic reviews. | Strongly Disagree | **Somewhat Disagree** | Somewhat Agree | Strongly Agree |
| --- | --- | --- | --- | --- |
| This overview adds value to the existing literature on this topic. | Strongly Disagree | Somewhat Disagree | **Somewhat Agree** | Strongly Agree |
| This overview would be useful to clinicians when compared to the individual systematic reviews included in the overview. | Strongly Disagree | Somewhat Disagree | **Somewhat Agree** | Strongly Agree |

| **First author** | **Campbell [4]** |
| --- | --- |
| Year of publication | 2016 |
| Clinical domain | Oncology |
| No. of included SRs | 4 |
| No. of included primary studies | Not reported |
| Type of eligible primary studies | Not specified |
| Population, Disease/Condition | Patients with cancer |
| Intervention: Group of drugs, Substance(s) | Cytostatic agents: Irinotecan  Exposure of interest: “Any form of germline polymorphism (that is, single-nucleotide polymorphisms, short tandem repeats and deletions)” |
| Comparator | Not specified |
| Adverse Event Outcome(s) of interest | Any irinotecan-induced toxicities, adverse events or side effects |
| Primary research question (as stated in the last sentence or paragraph of the introduction- or background-section, plus information given elsewhere, if more meaningful) | Introduction: “we have undertaken an umbrella review with the aim of identifying and synthesising the findings of all systematic reviews that have investigated associations between germline variations and irinotecan-induced toxicity in cancer patients. This allowed the identification of all polymorphisms, populations and contexts where biomarkers for irinotecan-induced toxicity have been validated, and are potentially ready for clinical application as risk factors to inform personalised medicine.” |

| This overview generates new knowledge not previously known from existing systematic reviews. | Strongly Disagree | Somewhat Disagree | **Somewhat Agree** | Strongly Agree |
| --- | --- | --- | --- | --- |
| This overview adds value to the existing literature on this topic. | Strongly Disagree | Somewhat Disagree | **Somewhat Agree** | Strongly Agree |
| This overview would be useful to clinicians when compared to the individual systematic reviews included in the overview. | Strongly Disagree | Somewhat Disagree | **Somewhat Agree** | Strongly Agree |

| **First author** | **Campbell [5]** |
| --- | --- |
| Year of publication | 2016 |
| Clinical domain | Oncology |
| No. of included SRs | 3 |
| No. of included primary studies | Not reported |
| Type of eligible primary studies | Not specified |
| Population, Disease/Condition | Patients with cancer |
| Intervention: Group of drugs, Substance(s) | Cytostatic agents: Methotrexate  Exposure of interest: “Any form of germline polymorphism [i.e. single nucleotide polymorphisms (SNPs), short tandem repeats, and deletions], whether detected by andidate gene analysis or GWAS.“ |
| Comparator | Not specified |
| Adverse Event Outcome(s) of interest | Any MTX-induced toxicities, adverse events or side effects |
| Primary research question (as stated in the last sentence or paragraph of the introduction- or background-section, plus information given elsewhere, if more meaningful) | Introduction: “The objective was to identify and synthesize the findings of all systematic reviews that have investigated associations between germline variations in cancer patients and MTX-induced toxicity to ensure the identification of all polymorphisms, populations, and contexts where biomarkers for MTX-induced toxicity have been validated, and are potentially ready for clinical application as risk factors to inform personalized medicine.” |

| This overview generates new knowledge not previously known from existing systematic reviews. | Strongly Disagree | **Somewhat Disagree** | Somewhat Agree | Strongly Agree |
| --- | --- | --- | --- | --- |
| This overview adds value to the existing literature on this topic. | Strongly Disagree | Somewhat Disagree | **Somewhat Agree** | Strongly Agree |
| This overview would be useful to clinicians when compared to the individual systematic reviews included in the overview. | Strongly Disagree | Somewhat Disagree | **Somewhat Agree** | Strongly Agree |

| **First author** | **Cates [6]** |
| --- | --- |
| Year of publication | 2014 |
| Clinical domain | Pneumology |
| No. of included SRs | 6 |
| No. of included primary studies | Not reported |
| Type of eligible primary studies | Randomized controlled trials |
| Population, Disease/Condition | Adults and adolescents (over the age of 12) with asthma |
| Intervention: Group of drugs, Substance(s) | Long-acting-beta-2-agonists (inhaled, either as monotherapy or as combination therapy): Formoterol, Salmeterol |
| Comparator | Formoterol/Salmeterol vs. Placebo, Formoterol vs. Salmeterol (alone and in combination with ICS), Formoterol plus ICS vs. same dose of ICS only, Salmeterol plus ICS vs. same dose of ICS only, Formoterol plus ICS vs. Salmeterol plus ICS |
| Adverse Event Outcome(s) of interest | Death of any cause, asthma-related serious deaths, number of participants with non-fatal serious adverse event of any cause or asthma-related non-fatal serious adverse event |
| Primary research question (as stated in the last sentence or paragraph of the introduction- or background-section, plus information given elsewhere, if more meaningful) | Objectives: “To assess the risk of serious adverse events in adults with asthma treated with regular maintenance formoterol or salmeterol compared with placebo, or when randomly assigned in combination with regular ICS, compared with the same dose of ICS.” |

| This overview generates new knowledge not previously known from existing systematic reviews. | Strongly Disagree | Somewhat Disagree | Somewhat Agree | **Strongly Agree** |
| --- | --- | --- | --- | --- |
| This overview adds value to the existing literature on this topic. | Strongly Disagree | Somewhat Disagree | **Somewhat Agree** | Strongly Agree |
| This overview would be useful to clinicians when compared to the individual systematic reviews included in the overview. | Strongly Disagree | **Somewhat Disagree** | Somewhat Agree | Strongly Agree |

| **First author** | **Cates [7]** |
| --- | --- |
| Year of publication | 2012 |
| Clinical domain | Pneumology |
| No. of included SRs | 6 |
| No. of included primary studies | 22 |
| Type of eligible primary studies | Randomized controlled trials |
| Population, Disease/Condition | Children with asthma |
| Intervention: Group of drugs, Substance(s) | Long-acting-beta-2-agonists (inhaled, either as monotherapy or as combination therapy): Formoterol, Salmeterol |
| Comparator | Formoterol/Salmeterol vs. Placebo, Formoterol vs. Salmeterol (alone and in combination with ICS), Formoterol plus ICS vs. same dose of ICS only, Salmeterol plus ICS vs. same dose of ICS only, Formoterol plus ICS vs. Salmeterol plus ICS |
| Adverse Event Outcome(s) of interest | Death of any cause, asthma-related serious deaths, number of participants with non-fatal serious adverse event of any cause or asthma-related non-fatal serious adverse event |
| Primary research question (as stated in the last sentence or paragraph of the introduction- or background-section, plus information given elsewhere, if more meaningful) | Objectives: “We have used the paediatric trial results from Cochrane systematic reviews to assess the safety of regular formoterol or salmeterol, either as monotherapy or as combination therapy, in children with asthma.” |

| This overview generates new knowledge not previously known from existing systematic reviews. | Strongly Disagree | Somewhat Disagree | Somewhat Agree | **Strongly Agree** |
| --- | --- | --- | --- | --- |
| This overview adds value to the existing literature on this topic. | Strongly Disagree | Somewhat Disagree | Somewhat Agree | **Strongly Agree** |
| This overview would be useful to clinicians when compared to the individual systematic reviews included in the overview. | Strongly Disagree | Somewhat Disagree | Somewhat Agree | **Strongly Agree** |

| **First author** | **Chen [8]** |
| --- | --- |
| Year of publication | 2016 |
| Clinical domain | Oncology |
| No. of included SRs | 42 |
| No. of included primary studies | Not reported |
| Type of eligible primary studies | Randomized controlled trials, observational studies, case series |
| Population, Disease/Condition | Patients with any disease |
| Intervention: Group of drugs, Substance(s) | Tumor necrosis facter alpha inhibitors: Adalimumab, Certolizumab pegol, Etanercept, Golilmumab, Infliximab |
| Comparator | Placebo, disease-modifying antirheumatic drugs, or any other effective drug (if a study had a control group) |
| Adverse Event Outcome(s) of interest | Any type of cancer |
| Primary research question (as stated in the last sentence or paragraph of the introduction- or background-section, plus information given elsewhere, if more meaningful) | Introduction: “In order to better understand the malignancy risk of anti-TNFα agents, we systematically performed this overview of systematic reviews and meta-analyses.”  Abstract: “The objective of the study is to systematically review the malignancy risk of anti-tumor necrosis factor alpha (anti-TNFα) agents.” |

| This overview generates new knowledge not previously known from existing systematic reviews. | Strongly Disagree | **Somewhat Disagree** | Somewhat Agree | Strongly Agree |
| --- | --- | --- | --- | --- |
| This overview adds value to the existing literature on this topic. | Strongly Disagree | Somewhat Disagree | **Somewhat Agree** | Strongly Agree |
| This overview would be useful to clinicians when compared to the individual systematic reviews included in the overview. | Strongly Disagree | Somewhat Disagree | **Somewhat Agree** | Strongly Agree |

| **First author** | **Dragioti [9]** |
| --- | --- |
| Year of publication | 2019 |
| Clinical domain | Psychiatry |
| No. of included SRs | 45 |
| No. of included primary studies | Not reported |
| Type of eligible primary studies | Observational studies |
| Population, Disease/Condition | Any population (any age) with no medical condition restrictions |
| Intervention: Group of drugs, Substance(s) | Antidepressants |
| Comparator | Not specified |
| Adverse Event Outcome(s) of interest | Any adverse health outcome |
| Primary research question (as stated in the last sentence or paragraph of the introduction- or background-section, plus information given elsewhere, if more meaningful) | Introduction: ” In this umbrella review,we graded the evidence from published meta-analyses of observational studies. These studies tested the association between antidepressant use and risk of adverse health outcomes.” |

| This overview generates new knowledge not previously known from existing systematic reviews. | Strongly Disagree | Somewhat Disagree | **Somewhat Agree** | Strongly Agree |
| --- | --- | --- | --- | --- |
| This overview adds value to the existing literature on this topic. | Strongly Disagree | Somewhat Disagree | **Somewhat Agree** | Strongly Agree |
| This overview would be useful to clinicians when compared to the individual systematic reviews included in the overview. | Strongly Disagree | **Somewhat Disagree** | Somewhat Agree | Strongly Agree |

| **First author** | **Els [10]** |
| --- | --- |
| Year of publication | 2017 |
| Clinical domain | Pain medicine |
| No. of included SRs | 16 |
| No. of included primary studies | 61 |
| Type of eligible primary studies | Randomized controlled studies |
| Population, Disease/Condition | Adults (aged 18 or older) with chronic non-cancer pain due to any condition |
| Intervention: Group of drugs, Substance(s) | Opioids (medium- (two weeks or longer) and long-term (two months or longer) use) |
| Comparator | Placebo or non-opioid treatment |
| Adverse Event Outcome(s) of interest | Number of participants with any (serious) adverse event, withdrawal due to adverse event, death, specific adverse event outcomes (24 different prespecified outcomes) |
| Primary research question (as stated in the last sentence or paragraph of the introduction- or background-section, plus information given elsewhere, if more meaningful) | Objectives: “To provide an overview of the occurrence and nature of adverse events associated with any opioid agent (any dose, frequency, or route of administration) used on a medium- or long-term basis for the treatment of CNCP in adults.” |

| This overview generates new knowledge not previously known from existing systematic reviews. | Strongly Disagree | Somewhat Disagree | **Somewhat Agree** | Strongly Agree |
| --- | --- | --- | --- | --- |
| This overview adds value to the existing literature on this topic. | Strongly Disagree | Somewhat Disagree | **Somewhat Agree** | Strongly Agree |
| This overview would be useful to clinicians when compared to the individual systematic reviews included in the overview. | Strongly Disagree | Somewhat Disagree | **Somewhat Agree** | Strongly Agree |

| **First author** | **Fernandes [11]** |
| --- | --- |
| Year of publication | 2014 |
| Clinical domain | Pneumology, asthma |
| No. of included SRs | 7 |
| No. of included primary studies | 88 |
| Type of eligible primary studies | Not specified |
| Population, Disease/Condition | Children (aged 0-18 years) with acute asthma, preschool wheezing, bronchiolitis, croup, pharyngitis/tonsillitis, or pneumonia |
| Intervention: Group of drugs, Substance(s) | Systemic corticosteroids (short-term (<two weeks) use) |
| Comparator | Placebo |
| Adverse Event Outcome(s) of interest | Death, hospital admission, length of stay in hospital, relapse leading to hospitalization, and specific adverse events (gastrointestinal bleeding and abdominal pain, behavioural issues, hypertension) |
| Primary research question (as stated in the last sentence or paragraph of the introduction- or background-section, plus information given elsewhere, if more meaningful) | Objectives: “Our objective was to examine clinically relevant safety outcomes related to acute single or recurrent short-term (<2 weeks) systemic corticosteroid use across reviews from a group of acute respiratory conditions.” |

| This overview generates new knowledge not previously known from existing systematic reviews. | Strongly Disagree | Somewhat Disagree | Somewhat Agree | **Strongly Agree** |
| --- | --- | --- | --- | --- |
| This overview adds value to the existing literature on this topic. | Strongly Disagree | Somewhat Disagree | Somewhat Agree | **Strongly Agree** |
| This overview would be useful to clinicians when compared to the individual systematic reviews included in the overview. | Strongly Disagree | Somewhat Disagree | Somewhat Agree | **Strongly Agree** |

| **First author** | **Gatti [12]** |
| --- | --- |
| Year of publication | 2020 |
| Clinical domain | Infectiology |
| No. of included SRs | 7 |
| No. of included primary studies | Not reported |
| Type of eligible primary studies | Randomized controlled trials, observational studies |
| Population, Disease/Condition | Not restricted |
| Intervention: Group of drugs, Substance(s) | Antibiotics: Fluoroquinolones |
| Comparator | Not specified |
| Adverse Event Outcome(s) of interest | Collagen-associated adverse events (three prespecified outcomes), neuropsychiatric toxicity, fluoroquinolone-associated disability |
| Primary research question (as stated in the last sentence or paragraph of the introduction- or background-section, plus information given elsewhere, if more meaningful) | Introduction: “In this umbrella review, we critically appraised current literature on emerging fluoroquinolone-related toxicities (namely collagen-associated AEs, neuropsychiatric toxicity and fluoroquinolone-associated disability), by assessing the quality and credibility of the evidence, in order to support clinicians in decision making in different clinical scenarios.“ |

| This overview generates new knowledge not previously known from existing systematic reviews. | Strongly Disagree | Somewhat Disagree | Somewhat Agree | **Strongly Agree** |
| --- | --- | --- | --- | --- |
| This overview adds value to the existing literature on this topic. | Strongly Disagree | Somewhat Disagree | Somewhat Agree | **Strongly Agree** |
| This overview would be useful to clinicians when compared to the individual systematic reviews included in the overview. | Strongly Disagree | Somewhat Disagree | Somewhat Agree | **Strongly Agree** |

| **First author** | **Grootens [13]** |
| --- | --- |
| Year of publication | 2018 |
| Clinical domain | Psychiatry |
| No. of included SRs | 18 |
| No. of included primary studies | Not reported |
| Type of eligible primary studies | Not specified |
| Population, Disease/Condition | Patients (non-pregnant, non-post partum) with a psychiatric disorder |
| Intervention: Group of drugs, Substance(s) | Non-lithium mood stabilizers: Valproate, Lamotrigine, Topiramate, Carbamazepine |
| Comparator | Not specified |
| Adverse Event Outcome(s) of interest | Weight change |
| Primary research question (as stated in the last sentence or paragraph of the introduction- or background-section, plus information given elsewhere, if more meaningful) | Introduction: “The objectives of the present study then are to give an overview of the literature about unwanted weight changes associated with the use of antiepileptic mood stabilizers in bipolar disorder and provide a summary and discussion of evidence-based practices for the prevention, monitoring, and treatment of these side effects. Based on these, we will formulate recommendations to guide patients and clinicians in their search for practical solutions to weight changes induced by antiepileptic mood stabilizers.” |

| This overview generates new knowledge not previously known from existing systematic reviews. | Strongly Disagree | Somewhat Disagree | **Somewhat Agree** | Strongly Agree |
| --- | --- | --- | --- | --- |
| This overview adds value to the existing literature on this topic. | Strongly Disagree | Somewhat Disagree | Somewhat Agree | **Strongly Agree** |
| This overview would be useful to clinicians when compared to the individual systematic reviews included in the overview. | Strongly Disagree | **Somewhat Disagree** | Somewhat Agree | Strongly Agree |

| **First author** | **Ijaz [14]** |
| --- | --- |
| Year of publication | 2018 |
| Clinical domain | Psychiatry |
| No. of included SRs | 12 |
| No. of included primary studies | Not reported |
| Type of eligible primary studies | Not specified |
| Population, Disease/Condition | Adults with schizophrenia |
| Intervention: Group of drugs, Substance(s) | Antipsychotics Exposure of interest: Antipsychotic polypharmacy |
| Comparator | Not specified |
| Adverse Event Outcome(s) of interest | Metabolic syndrome (defined as diabetes, hypertension, or hyperlipidaemia) |
| Primary research question (as stated in the last sentence or paragraph of the introduction- or background-section, plus information given elsewhere, if more meaningful) | Background: “To conduct a review of published systematic reviews to assess the current state of the evidence on the association between antipsychotic polypharmacy (APP) used for the management of schizophrenia and metabolic syndrome (defined as diabetes, hypertension, or hyperlipidaemia).” |

| This overview generates new knowledge not previously known from existing systematic reviews. | Strongly Disagree | Somewhat Disagree | Somewhat Agree | **Strongly Agree** |
| --- | --- | --- | --- | --- |
| This overview adds value to the existing literature on this topic. | Strongly Disagree | Somewhat Disagree | Somewhat Agree | **Strongly Agree** |
| This overview would be useful to clinicians when compared to the individual systematic reviews included in the overview. | Strongly Disagree | Somewhat Disagree | Somewhat Agree | **Strongly Agree** |

| **First author** | **Ioannidis [15]** |
| --- | --- |
| Year of publication | 2013 |
| Clinical domain | Oncology |
| No. of included SRs | 74 |
| No. of included primary studies | Not reported |
| Type of eligible primary studies | Randomized controlled trials, observational studies |
| Population, Disease/Condition | Not restricted |
| Intervention: Group of drugs, Substance(s) | Common medications that have been associated with increased cancer risk (defined as medications where there is at least one meta-analysis of randomized controlled trials that has claimed any nominally statistically significant increased cancer risk) |
| Comparator | Not specified |
| Adverse Event Outcome(s) of interest | Any type of cancer |
| Primary research question (as stated in the last sentence or paragraph of the introduction- or background-section, plus information given elsewhere, if more meaningful) | Introduction: “In this umbrella review, we aimed to collect systematically and critically reassess the results of meta-analyses of common medications that have been associated with increased cancer risk. We aimed to juxtapose the results of meta-analyses on similar drugs and cancer types to see how much they agree or disagree, to understand why disagreements may have arisen, and try to decipher eventually the presence or absence of cancer risks for these pharmacological and biological agents. [...] We aimed to see how strong and consistent the evidence was for these claims of significant cancer risks across different types of study designs and different meta-analyses on the same or similar topic.” |

| This overview generates new knowledge not previously known from existing systematic reviews. | Strongly Disagree | Somewhat Disagree | Somewhat Agree | **Strongly Agree** |
| --- | --- | --- | --- | --- |
| This overview adds value to the existing literature on this topic. | Strongly Disagree | Somewhat Disagree | Somewhat Agree | **Strongly Agree** |
| This overview would be useful to clinicians when compared to the individual systematic reviews included in the overview. | Strongly Disagree | Somewhat Disagree | Somewhat Agree | **Strongly Agree** |

| **First author** | **Lu [16]** |
| --- | --- |
| Year of publication | 2019 |
| Clinical domain | Orthopedics, Rheumatology, Endocrinology |
| No. of included SRs | 8 |
| No. of included primary studies | Not reported |
| Type of eligible primary studies | Randomized controlled trials, observational studies |
| Population, Disease/Condition | Patients with osteoporosis |
| Intervention: Group of drugs, Substance(s) | Bisphosphonates (as single agent or in combination regimens, long-term use (not defined)): Alendronate, Ibandronate, Etidronate, Risedronate, Zoledronic acid |
| Comparator | Placebo or any other drug |
| Adverse Event Outcome(s) of interest | Carcinogenity, atypical fracture, prolonged healing time of fracture, osteonecrosis of jaw |
| Primary research question (as stated in the last sentence or paragraph of the introduction- or background-section, plus information given elsewhere, if more meaningful) | Introduction: “[…] we decided to conduct an overview of systematic reviews to evaluate the risk of rare serious adverse effects (cancer, atypical fracture, ONJ and fracture union time) of long‐term use of BPs for the treatment of osteoporosis.” |

| This overview generates new knowledge not previously known from existing systematic reviews. | Strongly Disagree | **Somewhat Disagree** | Somewhat Agree | Strongly Agree |
| --- | --- | --- | --- | --- |
| This overview adds value to the existing literature on this topic. | Strongly Disagree | Somewhat Disagree | **Somewhat Agree** | Strongly Agree |
| This overview would be useful to clinicians when compared to the individual systematic reviews included in the overview. | Strongly Disagree | Somewhat Disagree | **Somewhat Agree** | Strongly Agree |

| **First author** | **Macias Saint-Gerons [17]** |
| --- | --- |
| Year of publication | 2020 |
| Clinical domain | Vaccination, Pregnancy |
| No. of included SRs | 17 |
| No. of included primary studies | Not reported |
| Type of eligible primary studies | Controlled studies (either randomized or non-randomized) |
| Population, Disease/Condition | Pregnant women |
| Intervention: Group of drugs, Substance(s) | Vaccinations: Hepatitis A/B, Influenza (incl. H1N1), Menigococcal disease, Poliomyelitis, Rabies, Tetanus/Diphtheria/Pertussis(incl. Tdap), Yellow fever |
| Comparator | Not specified |
| Adverse Event Outcome(s) of interest | Neonatal death, neonatal infection, chorioamnionitis, congenital anomalies, microcephaly, preterm birth (<37 weeks), abortion, stillbirth, low birth weight (<2500 grams), maternal death, small for gestational age |
| Primary research question (as stated in the last sentence or paragraph of the introduction- or background-section, plus information given elsewhere, if more meaningful) | Introduction: “We conducted an overview of systematic reviews reporting data on safety after immunization with recommended vaccines during pregnancy in the Maternal and Neonatal Immunization Field Guide for Latin America and the Caribbean.” |

| This overview generates new knowledge not previously known from existing systematic reviews. | Strongly Disagree | Somewhat Disagree | **Somewhat Agree** | Strongly Agree |
| --- | --- | --- | --- | --- |
| This overview adds value to the existing literature on this topic. | Strongly Disagree | Somewhat Disagree | Somewhat Agree | **Strongly Agree** |
| This overview would be useful to clinicians when compared to the individual systematic reviews included in the overview. | Strongly Disagree | Somewhat Disagree | **Somewhat Agree** | Strongly Agree |

| **First author** | **Mattishent [18]** |
| --- | --- |
| Year of publication | 2014 |
| Clinical domain | Pneumology |
| No. of included SRs | 5 |
| No. of included primary studies | Not reported |
| Type of eligible primary studies | Not specified |
| Population, Disease/Condition | Not clearly specified (most likely COPD and asthma) |
| Intervention: Group of drugs, Substance(s) | Corticosteroids (inhaled) |
| Comparator | Not specified |
| Adverse Event Outcome(s) of interest | Fractures, pneumonia, cataract, hyperglycaemia |
| Primary research question (as stated in the last sentence or paragraph of the introduction- or background-section, plus information given elsewhere, if more meaningful) | Introduction: “our objective was to evaluate and critically appraise up-to-date evidence regarding the risk of ICS use in COPD patients, so that practitioners and patients can make informed decisions and optimise the management of COPD. We chose to focus on fractures, pneumonia, cataracts and hyperglycaemia because these are of particular relevance to older patients.” |

| This overview generates new knowledge not previously known from existing systematic reviews. | Strongly Disagree | Somewhat Disagree | Somewhat Agree | **Strongly Agree** |
| --- | --- | --- | --- | --- |
| This overview adds value to the existing literature on this topic. | Strongly Disagree | Somewhat Disagree | Somewhat Agree | **Strongly Agree** |
| This overview would be useful to clinicians when compared to the individual systematic reviews included in the overview. | Strongly Disagree | Somewhat Disagree | Somewhat Agree | **Strongly Agree** |

| **First author** | **Moore [19]** |
| --- | --- |
| Year of publication | 2015 |
| Clinical domain | Pain medicine |
| No. of included SRs | 39 |
| No. of included primary studies | 399 |
| Type of eligible primary studies | Randomized controlled trials |
| Population, Disease/Condition | Adults (aged 15 or older), acute postoperative pain |
| Intervention: Group of drugs, Substance(s) | Analgesics (oral, single dose) |
| Comparator | Placebo |
| Adverse Event Outcome(s) of interest | Number of participants with at least one (serious) adverse event, death |
| Primary research question (as stated in the last sentence or paragraph of the introduction- or background-section, plus information given elsewhere, if more meaningful) | Objectives: “To provide an overview of adverse event rates associated with single dose oral analgesics, compared with placebo, for acute postoperative pain in adults.” |

| This overview generates new knowledge not previously known from existing systematic reviews. | Strongly Disagree | Somewhat Disagree | **Somewhat Agree** | Strongly Agree |
| --- | --- | --- | --- | --- |
| This overview adds value to the existing literature on this topic. | Strongly Disagree | Somewhat Disagree | **Somewhat Agree** | Strongly Agree |
| This overview would be useful to clinicians when compared to the individual systematic reviews included in the overview. | Strongly Disagree | Somewhat Disagree | **Somewhat Agree** | Strongly Agree |

| **First author** | **Onasanya [20]** |
| --- | --- |
| Year of publication | 2016 |
| Clinical domain | Endocrinology |
| No. of included SRs | 7 |
| No. of included primary studies | 94 |
| Type of eligible primary studies | Randomized controlled trials |
| Population, Disease/Condition | Adult men (aged 18 or older) |
| Intervention: Group of drugs, Substance(s) | Testosterone |
| Comparator | Not specified |
| Adverse Event Outcome(s) of interest | Any cardiovascular event |
| Primary research question (as stated in the last sentence or paragraph of the introduction- or background-section, plus information given elsewhere, if more meaningful) | Introduction: “We did an overview of published systematic reviews of randomised controlled trials and qualitatively assessed the association between exogenous testosterone and cardiovascular risks. In addition to investigating the direction and magnitude of the primary association of interest, we compared and contrasted study characteristics, analytic methods, included trials, key findings, and methodological quality of each synthesis.” |

| This overview generates new knowledge not previously known from existing systematic reviews. | Strongly Disagree | Somewhat Disagree | **Somewhat Agree** | Strongly Agree |
| --- | --- | --- | --- | --- |
| This overview adds value to the existing literature on this topic. | Strongly Disagree | Somewhat Disagree | **Somewhat Agree** | Strongly Agree |
| This overview would be useful to clinicians when compared to the individual systematic reviews included in the overview. | Strongly Disagree | Somewhat Disagree | **Somewhat Agree** | Strongly Agree |

| **First author** | **Ozbilen [21]** |
| --- | --- |
| Year of publication | 2009 |
| Clinical domain | Psychiatry |
| No. of included SRs | 54 |
| No. of included primary studies | 177 |
| Type of eligible primary studies | Randomized controlled trials |
| Population, Disease/Condition | Patients with schizophrenia, schizophreniform disorder, or schizotypal disorder |
| Intervention: Group of drugs, Substance(s) | Antipsychotics |
| Comparator | Not specified |
| Adverse Event Outcome(s) of interest | Anticholinergic effects (nine prespecified outcomes) |
| Primary research question (as stated in the last sentence or paragraph of the introduction- or background-section, plus information given elsewhere, if more meaningful) | Aims: “To estimate the prevalence of anticholinergic effects for people with schizophrenia allocated antipsychotic drugs within randomized trials.” |

| This overview generates new knowledge not previously known from existing systematic reviews. | Strongly Disagree | Somewhat Disagree | Somewhat Agree | **Strongly Agree** |
| --- | --- | --- | --- | --- |
| This overview adds value to the existing literature on this topic. | Strongly Disagree | Somewhat Disagree | Somewhat Agree | **Strongly Agree** |
| This overview would be useful to clinicians when compared to the individual systematic reviews included in the overview. | Strongly Disagree | Somewhat Disagree | Somewhat Agree | **Strongly Agree** |

| **First author** | **Pelletier [22]** |
| --- | --- |
| Year of publication | 2021 |
| Clinical domain | Diabetology |
| No. of included SRs | 47 |
| No. of included primary studies | Not reported |
| Type of eligible primary studies | Randomized controlled trials, observational studies |
| Population, Disease/Condition | Patients with type 2 diabetes |
| Intervention: Group of drugs, Substance(s) | Sodium-glucose transport protein 2 Inhibitors |
| Comparator | Not specified |
| Adverse Event Outcome(s) of interest | Total adverse events, serious adverse events, withdrawals due to adverse events, infections, volume depletion-related events, acute kidney injury, bone fractures, diabetic ketoacidosis, lower limb amputations, cancer, other notable adverse events |
| Primary research question (as stated in the last sentence or paragraph of the introduction- or background-section, plus information given elsewhere, if more meaningful) | Introduction: “we conducted an overview of systematic reviews, adapted from Cochrane Overviews, to provide clinicians, policy-makers and clinical guideline developers with a critical appraisal and summary of the best available evidence assessing the safety of SGLT-2 inhibitors used in the treatment of type 2 diabetes.” |

| This overview generates new knowledge not previously known from existing systematic reviews. | Strongly Disagree | **Somewhat Disagree** | Somewhat Agree | Strongly Agree |
| --- | --- | --- | --- | --- |
| This overview adds value to the existing literature on this topic. | Strongly Disagree | Somewhat Disagree | **Somewhat Agree** | Strongly Agree |
| s overview would be useful to clinicians when compared to the individual systematic reviews included in the overview. | Strongly Disagree | Somewhat Disagree | **Somewhat Agree** | Strongly Agree |

| **First author** | **Pelletier [23]** |
| --- | --- |
| Year of publication | 2020 |
| Clinical domain | Diabetology, Oncology |
| No. of included SRs | 8 |
| No. of included primary studies | Not reported |
| Type of eligible primary studies | Randomized controlled trials, observational studies |
| Population, Disease/Condition | Patients with type 2 diabetes |
| Intervention: Group of drugs, Substance(s) | Sodium-glucose transport protein 2 Inhibitors |
| Comparator | Not specified |
| Adverse Event Outcome(s) of interest | Any type of cancer |
| Primary research question (as stated in the last sentence or paragraph of the introduction- or background-section, plus information given elsewhere, if more meaningful) | Introduction: “We aimed to summarize evidence from and assess the quality of published quantitative systematic reviews evaluating the cancer risk associated with SGLT-2 inhibitor use in the treatment of type 2 diabetes.” |

| This overview generates new knowledge not previously known from existing systematic reviews. | Strongly Disagree | Somewhat Disagree | **Somewhat Agree** | Strongly Agree |
| --- | --- | --- | --- | --- |
| This overview adds value to the existing literature on this topic. | Strongly Disagree | Somewhat Disagree | **Somewhat Agree** | Strongly Agree |
| This overview would be useful to clinicians when compared to the individual systematic reviews included in the overview. | Strongly Disagree | Somewhat Disagree | **Somewhat Agree** | Strongly Agree |

| **First author** | **Salvo [24]** |
| --- | --- |
| Year of publication | 2011 |
| Clinical domain | Cardiology, Gastroenterology |
| No. of included SRs | 29 |
| No. of included primary studies | Not reported |
| Type of eligible primary studies | Randomized controlled trials |
| Population, Disease/Condition | Adults |
| Intervention: Group of drugs, Substance(s) | Non-steroidal anti-inflammatory drugs (either tNSAIDs, Coxibs, or Aspirin at 100mg/day) |
| Comparator | Not specified |
| Adverse Event Outcome(s) of interest | Gastrointestinal adverse events (four prespecified outcomes), cardiovascular adverse events (seven prespecified outcomes) |
| Primary research question (as stated in the last sentence or paragraph of the introduction- or background-section, plus information given elsewhere, if more meaningful) | Introduction: “To synthesize the information on absolute risks of GI and CV adverse events associated with the use of individual NSAIDs in RCTs, and to identify knowledge gaps in the safety evaluation of these drugs, a systematic quantitative review of the MAs on NSAID RCTs was conducted.” |

| This overview generates new knowledge not previously known from existing systematic reviews. | Strongly Disagree | **Somewhat Disagree** | Somewhat Agree | Strongly Agree |
| --- | --- | --- | --- | --- |
| This overview adds value to the existing literature on this topic. | Strongly Disagree | **Somewhat Disagree** | Somewhat Agree | Strongly Agree |
| This overview would be useful to clinicians when compared to the individual systematic reviews included in the overview. | Strongly Disagree | Somewhat Disagree | **Somewhat Agree** | Strongly Agree |

| **First author** | **Thulliez [25]** |
| --- | --- |
| Year of publication | 2018 |
| Clinical domain | Ophthalmology |
| No. of included SRs | 21 |
| No. of included primary studies | Not reported |
| Type of eligible primary studies | Randomized controlled trials, observational studies |
| Population, Disease/Condition | Patients with age-related macular degeneration, diabetic macular edema, or retinal vein occlusion |
| Intervention: Group of drugs, Substance(s) | Anti-vascular endothelial growth factors (intravitreal): Ranibizumab, Bevacizumab, Aflibercept, Pegaptanib |
| Comparator | Not restricted |
| Adverse Event Outcome(s) of interest | Systemic adverse events (partially pre-specified) |
| Primary research question (as stated in the last sentence or paragraph of the introduction- or background-section, plus information given elsewhere, if more meaningful) | Introduction: “The objective of our work was to establish an overall view of the evidence by explicitly and systematically reviewing all available evidence in systematic reviews and meta-analyses reporting systemic adverse events (SAEs) with intravitreal anti-VEGF(ranibizumab, bevacizumab, aflibercept, and pegaptanib).” |

| This overview generates new knowledge not previously known from existing systematic reviews. | Strongly Disagree | Somewhat Disagree | **Somewhat Agree** | Strongly Agree |
| --- | --- | --- | --- | --- |
| This overview adds value to the existing literature on this topic. | Strongly Disagree | Somewhat Disagree | Somewhat Agree | **Strongly Agree** |
| This overview would be useful to clinicians when compared to the individual systematic reviews included in the overview. | Strongly Disagree | Somewhat Disagree | **Somewhat Agree** | Strongly Agree |

| **First author** | **Uguz [26]** |
| --- | --- |
| Year of publication | 2020 |
| Clinical domain | Psychiatry, Pregnancy |
| No. of included SRs | 15 |
| No. of included primary studies | Not reported |
| Type of eligible primary studies | Not specified |
| Population, Disease/Condition | Pregnant women in the first trimester |
| Intervention: Group of drugs, Substance(s) | Antidepressants: Selective serotonin reuptake inhibitors |
| Comparator | Not specified |
| Adverse Event Outcome(s) of interest | Congenital anomalies |
| Primary research question (as stated in the last sentence or paragraph of the introduction- or background-section, plus information given elsewhere, if more meaningful) | Introduction: “This paper aims to systematically review the results of recent meta-analyses investigating the use of SSRIs during pregnancy and the risk of congenital anomalies. Since there is a large body of data in the literature regarding the perinatal safety of antidepressants, this review only focuses on the risk of congenital anomalies.” |

| This overview generates new knowledge not previously known from existing systematic reviews. | **Strongly Disagree** | Somewhat Disagree | Somewhat Agree | Strongly Agree |
| --- | --- | --- | --- | --- |
| This overview adds value to the existing literature on this topic. | Strongly Disagree | Somewhat Disagree | **Somewhat Agree** | Strongly Agree |
| This overview would be useful to clinicians when compared to the individual systematic reviews included in the overview. | Strongly Disagree | **Somewhat Disagree** | Somewhat Agree | Strongly Agree |

| **First author** | **Van Leeuwen [27]** |
| --- | --- |
| Year of publication | 2020 |
| Clinical domain | Oncology, Cardiology |
| No. of included SRs | 113 |
| No. of included primary studies | Not reported |
| Type of eligible primary studies | Randomized controlled trials, observational studies |
| Population, Disease/Condition | Patients with cancer |
| Intervention: Group of drugs, Substance(s) | Targeted cancer therapeutics: Monoclonal antibodies, Protein kinase inhibitors, Antineoplastic agents, Hormone antagonists and related agents, Immunomodulating agents |
| Comparator | Placebo, active comparator |
| Adverse Event Outcome(s) of interest | Cardiovascular adverse events (partially pre-specified) |
| Primary research question (as stated in the last sentence or paragraph of the introduction- or background-section, plus information given elsewhere, if more meaningful) | Introduction: “In this overview, we provide an accessible synthesis with which to inform clinicians in general practice, cardiology, and oncology, as well as patients, when managing cardiovascular health.”  Abstract: “We classified evidence of cardiovascular toxicity as sufficient, probable, possible, or indeterminate for specific cardiovascular outcomes based on statistical significance, study quality, and size.” |

| This overview generates new knowledge not previously known from existing systematic reviews. | Strongly Disagree | Somewhat Disagree | Somewhat Agree | **Strongly Agree** |
| --- | --- | --- | --- | --- |
| This overview adds value to the existing literature on this topic. | Strongly Disagree | Somewhat Disagree | **Somewhat Agree** | Strongly Agree |
| This overview would be useful to clinicians when compared to the individual systematic reviews included in the overview. | Strongly Disagree | Somewhat Disagree | **Somewhat Agree** | Strongly Agree |

**References**

[1] Abramowitz J, Thakkar P, Isa A, Truong A, Park C, Rosenfeld RM. Adverse Event Reporting for Proton Pump Inhibitor Therapy: An Overview of Systematic Reviews. Otolaryngol Head Neck Surg. 2016;155:547-54. <https://doi.org/10.1177/0194599816648298>

[2] Bonovas S, Pantavou K, Evripidou D, Bastiampillai AJ, Nikolopoulos GK, Peyrin-Biroulet L, et al. Safety of biological therapies in ulcerative colitis: An umbrella review of meta-analyses. Best Pract Res Clin Gastroenterol. 2018;32-33:43-7. <https://doi.org/10.1016/j.bpg.2018.05.005>

[3] Campbell JM, Bateman E, Peters M, Bowen JM, Keefe DM, Stephenson MD. Fluoropyrimidine and platinum toxicity pharmacogenetics: an umbrella review of systematic reviews and meta-analyses. Pharmacogenomics. 2016;17:435-51. <https://doi.org/10.2217/pgs.15.180>

[4] Campbell JM, Stephenson MD, Bateman E, Peters MD, Keefe DM, Bowen JM. Irinotecan-induced toxicity pharmacogenetics: an umbrella review of systematic reviews and meta-analyses. Pharmacogenomics J. 2017;17:21-8. <https://doi.org/10.1038/tpj.2016.58>

[5] Campbell JM, Bateman E, Stephenson MD, Bowen JM, Keefe DM, Peters MD. Methotrexate-induced toxicity pharmacogenetics: an umbrella review of systematic reviews and meta-analyses. Cancer Chemother Pharmacol. 2016;78:27-39. <https://doi.org/10.1007/s00280-016-3043-5>

[6] Cates CJ, Wieland LS, Oleszczuk M, Kew KM. Safety of regular formoterol or salmeterol in adults with asthma: an overview of Cochrane reviews. Cochrane Database Syst Rev. 2014:CD010314. <https://doi.org/10.1002/14651858.CD010314.pub2>

[7] Cates CJ, Oleszczuk M, Stovold E, Wieland LS. Safety of regular formoterol or salmeterol in children with asthma: an overview of Cochrane reviews. Cochrane Database Syst Rev. 2012;10:CD010005. <https://doi.org/10.1002/14651858.CD010005.pub2>

[8] Chen Y, Sun J, Yang Y, Huang Y, Liu G. Malignancy risk of anti-tumor necrosis factor alpha blockers: an overview of systematic reviews and meta-analyses. Clin Rheumatol. 2016;35:1-18. <https://doi.org/10.1007/s10067-015-3115-7>

[9] Dragioti E, Solmi M, Favaro A, Fusar-Poli P, Dazzan P, Thompson T, et al. Association of Antidepressant Use With Adverse Health Outcomes: A Systematic Umbrella Review. JAMA Psychiatry. 2019;76:1241-55. <https://doi.org/10.1001/jamapsychiatry.2019.2859>

[10] Els C, Jackson TD, Kunyk D, Lappi VG, Sonnenberg B, Hagtvedt R, et al. Adverse events associated with medium- and long-term use of opioids for chronic non-cancer pain: an overview of Cochrane Reviews. Cochrane Database Syst Rev. 2017;10:CD012509. <https://doi.org/10.1002/14651858.CD012509.pub2>

[11] Fernandes RM, Oleszczuk M, Woods CR, Rowe BH, Cates CJ, Hartling L. The Cochrane Library and safety of systemic corticosteroids for acute respiratory conditions in children: an overview of reviews. Evid Based Child Health. 2014;9:733-47. <https://doi.org/10.1002/ebch.1980>

[12] Gatti M, Bianchin M, Raschi E, De Ponti F. Assessing the association between fluoroquinolones and emerging adverse drug reactions raised by regulatory agencies: An umbrella review. Eur J Intern Med. 2020;75:60-70. <https://doi.org/10.1016/j.ejim.2020.01.009>

[13] Grootens KP, Meijer A, Hartong EG, Doornbos B, Bakker PR, Al Hadithy A, et al. Weight changes associated with antiepileptic mood stabilizers in the treatment of bipolar disorder. Eur J Clin Pharmacol. 2018;74:1485-9. <https://doi.org/10.1007/s00228-018-2517-2>

[14] Ijaz S, Bolea B, Davies S, Savovic J, Richards A, Sullivan S, et al. Antipsychotic polypharmacy and metabolic syndrome in schizophrenia: a review of systematic reviews. BMC Psychiatry. 2018;18:275. <https://doi.org/10.1186/s12888-018-1848-y>

[15] Ioannidis JP, Zhou Y, Chang CQ, Schully SD, Khoury MJ, Freedman AN. Potential increased risk of cancer from commonly used medications: an umbrella review of meta-analyses. Ann Oncol. 2014;25:16-23. <https://doi.org/10.1093/annonc/mdt372>

[16] Lu L, Lu L, Zhang J, Li J. Potential risks of rare serious adverse effects related to long-term use of bisphosphonates: An overview of systematic reviews. J Clin Pharm Ther. 2020;45:45-51. <https://doi.org/10.1111/jcpt.13056>

[17] Macias Saint-Gerons D, Sola Arnau I, De Mucio B, Arevalo-Rodriguez I, Aleman A, Castro JL, et al. Adverse events associated with the use of recommended vaccines during pregnancy: An overview of systematic reviews. Vaccine. 2021;39 Suppl 2:B12-B26. <https://doi.org/10.1016/j.vaccine.2020.07.048>

[18] Mattishent K, Thavarajah M, Blanco P, Gilbert D, Wilson AM, Loke YK. Meta-review: adverse effects of inhaled corticosteroids relevant to older patients. Drugs. 2014;74:539-47. <https://doi.org/10.1007/s40265-014-0202-z>

[19] Moore RA, Derry S, Aldington D, Wiffen PJ. Adverse events associated with single dose oral analgesics for acute postoperative pain in adults - an overview of Cochrane reviews. Cochrane Database Syst Rev. 2015:CD011407. <https://doi.org/10.1002/14651858.CD011407.pub2>

[20] Onasanya O, Iyer G, Lucas E, Lin D, Singh S, Alexander GC. Association between exogenous testosterone and cardiovascular events: an overview of systematic reviews. Lancet Diabetes Endocrinol. 2016;4:943-56. <https://doi.org/10.1016/S2213-8587(16)30215-7>

[21] Ozbilen M, Adams CE. Systematic overview of Cochrane reviews for anticholinergic effects of antipsychotic drugs. J Clin Psychopharmacol. 2009;29:141-6. <https://doi.org/10.1097/JCP.0b013e31819a91f1>

[22] Pelletier R, Ng K, Alkabbani W, Labib Y, Mourad N, Gamble JM. Adverse events associated with sodium glucose co-transporter 2 inhibitors: an overview of quantitative systematic reviews. Ther Adv Drug Saf. 2021;12:2042098621989134. <https://doi.org/10.1177/2042098621989134>

[23] Pelletier R, Ng K, Alkabbani W, Labib Y, Mourad N, Gamble JM. The association of sodium-glucose cotransporter 2 inhibitors with cancer: An overview of quantitative systematic reviews. Endocrinol Diabetes Metab. 2020;3:e00145. <https://doi.org/10.1002/edm2.145>

[24] Salvo F, Fourrier-Reglat A, Bazin F, Robinson P, Riera-Guardia N, Haag M, et al. Cardiovascular and gastrointestinal safety of NSAIDs: a systematic review of meta-analyses of randomized clinical trials. Clin Pharmacol Ther. 2011;89:855-66. <https://doi.org/10.1038/clpt.2011.45>

[25] Thulliez M, Angoulvant D, Pisella PJ, Bejan-Angoulvant T. Overview of Systematic Reviews and Meta-analyses on Systemic Adverse Events Associated With Intravitreal Anti-Vascular Endothelial Growth Factor Medication Use. JAMA Ophthalmol. 2018;136:557-66. <https://doi.org/10.1001/jamaophthalmol.2018.0002>

[26] Uguz F. Selective serotonin reuptake inhibitors and the risk of congenital anomalies: a systematic review of current meta-analyses. Expert Opin Drug Saf. 2020;19:1595-604. <https://doi.org/10.1080/14740338.2020.1832080>

[27] Van Leeuwen MT, Luu S, Gurney H, Brown MR, Pearson SA, Webber K, et al. Cardiovascular Toxicity of Targeted Therapies for Cancer: An Overview of Systematic Reviews. JNCI Cancer Spectr. 2020;4:pkaa076. <https://doi.org/10.1093/jncics/pkaa076>
